# Informal Caregiver Burden in Palliative Care and the Role of the Family Doctor: A Scoping Review

**DOI:** 10.3390/healthcare13080939

**Published:** 2025-04-19

**Authors:** Laura Lapa, Marta Cardoso, Francisca Rego

**Affiliations:** 1 Unidade de Saúde Familiar Nova Mateus, Unidade Local de Saúde de Trás-os-Montes e Alto Douro, 5000-577 Vila Real, Portugal; 2 Unidade de Saúde Familiar Régua, Unidade Local de Saúde de Trás-os-Montes e Alto Douro, 5050-275 Peso da Régua, Portugal; marta.cardoso@arsnorte.min-saude.pt; 3 Faculty of Medicine, University of Porto, 4200-319 Porto, Portugal; mfrego@med.up.pt

**Keywords:** caregiver burden, family physician, general practitioner, palliative care, primary care physicians

## Abstract

**Background/Objectives:** Caregivers play a central role in supporting patients in palliative care but often face significant challenges to their physical, emotional, social, and financial well-being. Family doctors are uniquely positioned to help alleviate this burden through early identification, targeted interventions, and coordinated care. This scoping review analyzed existing literature on caregiver burden in palliative care to explore the specific role of family doctors in identifying, preventing, and reducing this burden. **Methods**: A scoping review was carried out following the methodology set out by the Joanna Briggs Institute. The following databases were searched: PubMed, the Cochrane Library, Scopus, the National Institute for Health and Care Excellence, and the British Medical Journal. The search strategy was based on the use of the following keywords and Medical Subject Headings: “caregiver burden” AND “palliative care” AND (“family physician” OR “general practitioners” OR “primary care physicians”). The search was performed on 10 March 2024, with a time horizon between 2013 and 2023. **Results**: From 259 identified articles, 8 met the inclusion criteria. Key themes included factors influencing caregiver burden, strategies used by family doctors to mitigate it, and challenges in providing support. Family doctors play a crucial role in offering psychological support, educating caregivers on disease progression, and coordinating multidisciplinary care. **Conclusions**: The active involvement of family doctors significantly reduces caregiver burden by addressing emotional distress, improving communication, and ensuring care coordination. Key interventions include early distress screening, tailored education, and access to multidisciplinary networks. Strengthening their integration in palliative care teams is essential for optimizing patient and caregiver outcomes.

## 1. Introduction

Palliative care (PC) has a holistic and active approach to the management of serious, chronic, and progressive illnesses, with the primary objective of improving the quality of life of patients, their families, and caregivers. The central tenet of PC is the prevention and relief of suffering, achieved through the early identification and appropriate treatment of pain and other physical, psychological, social, and spiritual problems. This care should be applied throughout the course of the illness, in conjunction with modifying or curative therapies [1].

PC is provided by interdisciplinary teams that adhere to ethical principles and promote advanced care planning. The involvement of family members and informal caregivers is crucial, and their empowerment and support in managing bereavement are paramount. Respect for the patient’s autonomy and values is paramount, ensuring a personalized and dignified approach [2].

Informal caregivers, typically family members or close friends, assume a pivotal role in the care of patients, undertaking a wide range of responsibilities encompassing support for daily activities and the management of medical care within the domestic environment. This role presents significant challenges, impacting caregivers’ physical, emotional, social and financial well-being [3,4].

A significant proportion of patients express a desire to remain in their own homes until the end of their lives, a phenomenon that has given rise to the development of policies that promote home-based PC [5,6]. However, this option requires a high level of involvement from informal caregivers, who accumulate responsibilities such as the administration of medication, the provision of emotional support, and the management of medical decisions, while facing high levels of emotional overload [7,8].

The caregiver burden arises from an imbalance between the demands imposed by care and the available resources, affecting both the physical and psychological well-being of the caregiver [9]. Studies have indicated that many caregivers experience symptoms of anxiety and depression, as well as manifesting extreme tiredness, sleep disturbances, and a decline in general health [10].

The lack of adequate training and support has been demonstrated to exacerbate feelings of insecurity among caregivers, who often neglect their own needs in order to devote themselves to the patient [11]. Furthermore, challenges in family dynamics and the absence of external support contribute to mounting pressure on the primary caregivers, impeding the distribution of responsibilities in a balanced manner [12,13,14,15,16,17,18,19,20,21,22,23,24,25].

The field of Family Medicine (FM) is centered on the delivery of primary health care, assuming a pivotal role in the integration and coordination of care, with an emphasis on a comprehensive and continuous approach across the entire spectrum of patients’ lives [26]. In their professional capacity, family doctors (FD) are responsible for promoting health, preventing disease, and ensuring the most appropriate care plan for their patients, whether through the provision of curative care and/or PC.

### Review Questions

This scoping review aims to answer the following questions:

1. Primary question:
  – What does the literature say regarding the nature of the burden of the informal caregiver in PC, and what is the role of the FD in minimizing this burden?
2. Secondary questions:
  – What are the determining factors that influence the burden of informal caregivers in the context of PC?
  – What interventions implemented by the FD have been investigated in the literature to reduce the burden of informal caregivers in PC?
  – What are the main challenges and barriers encountered by the FD in providing support to informal caregivers in PC?

## 2. Methods

This scoping review was conducted in accordance with the methodology of the Joanna Briggs Institute [27].

During the conduct of this scoping review, five key steps were followed, namely, defining the research questions, searching for and identifying relevant studies, selecting the studies, systematically extracting the data, and drawing up a descriptive summary, accompanied by a structured presentation of the results obtained [27].

The participants eligible for inclusion in this review comprised two main groups: informal caregivers of PC patients and healthcare professionals, specifically FDs, who are involved in the provision of PC.

The review addressed several central concepts, including the burden of the informal caregiver, the strategies adopted by these caregivers, and the interventions implemented by the FD in the context of PC.

The studies that met the inclusion criteria were conducted within the framework of primary health care, which is characterized by the active involvement of the FD in the delivery of PC.

The review included a wide range of study types, encompassing quantitative, qualitative, systematic, and scoping reviews, to ensure a comprehensive exploration of the existing literature. The publication dates of the articles in question ranged from 2013 to 2023.

The inclusion criteria encompassed articles published in Portuguese, English, and Spanish.

The exclusion criteria established for this review involved the selection of studies that did not directly address the burden of the informal caregiver in the context of PC. Research that did not discuss the role or interventions of the FD in this context was also excluded.

The search strategy was implemented in the following databases: PubMed, the Cochrane Library, Scopus, the National Institute for Health and Care Excellence (NICE), and the British Medical Journal (BMJ). The search strategy was based on the following keywords and Medical Subject Headings (MeSH): “caregiver burden” AND “palliative care” AND (“family physician” OR “general practitioners” OR “primary care physicians”).

The selection of studies was conducted in two stages, based on a search carried out on 10 March 2024, in the aforementioned databases. In the initial stage, the titles and abstracts of the articles identified were analyzed based on the inclusion criteria previously established. Subsequently, the full texts of the studies selected in the first phase were assessed in detail to confirm their eligibility. The full collection of articles identified through this process was imported into the reference management software Mendeley version 1.19.8 for further analysis.

## 3. Results

### 3.1. Search Results

**Figure 1:**
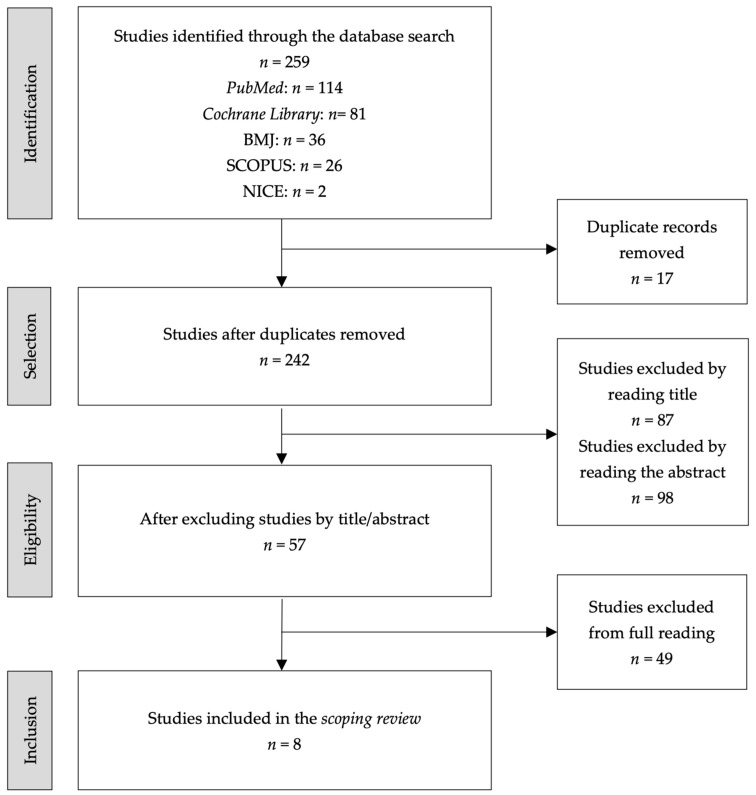
Flowchart of research results and study selection and inclusion process (PRISMA-ScR).

A total of 114 studies were identified in the PubMed database, 81 in the Cochrane Library, 36 in the BMJ, 26 in SCOPUS, and 2 in NICE, resulting in a total of 259 studies. Following the removal of 17 duplicate studies, 242 studies were selected for subsequent analysis. This initial analysis entailed a meticulous evaluation of the titles and abstracts, facilitating the screening of relevant studies for the research in question. Consequently, 184 studies were excluded. The subsequent stage entailed a full reading of the articles, which led to the inclusion of eight studies in the scoping review. The results of the review were presented in accordance with the Preferred Reporting Items for Systematic Reviews and Meta-Analyses extension for Scoping Reviews (PRISMA-ScR) guidelines, as illustrated in Figure 1 [27].

#### 3.1.1. Distribution by Year of Publication and Country

The studies included in this analysis were published between 2016 and 2023, with the oldest published in 2016 and the most recent in 2023. The year which saw the highest number of publications was 2021, with three studies, representing 37.5% of all studies (*n* = 3; 37.5%). With regard to geographical origin, the majority of studies were from Germany (*n* = 2; 25%) and Canada (*n* = 2; 25%).

#### 3.1.2. Distribution by Type of Approach

The majority of studies included in the analysis employed a qualitative approach (*n* = 5, 62.5%). Two studies presented quantitative results, consisting of a randomized clinical trial and a prospective cohort study (*n* = 2; 25%). In addition, a scoping review was included (*n* = 1; 12.5%), with the aim of analyzing the existing literature and providing a comprehensive overview of the topic under investigation.

The qualitative studies generally had small samples of informal caregivers (*n* ≤ 35) and family doctors (*n* ≤ 18). With regard to the quantitative studies, it is worth mentioning that the samples ranged from 54 to 100 informal caregivers. It is noteworthy that one of the studies focused exclusively on the perspective of informal caregivers. The studies included address informal caregivers of patients with oncological and non-oncological diseases (diabetes, dementia, and other chronic diseases).

The eight articles selected for inclusion in the review are enumerated in Table 1, accompanied by the following details: the title of the work, the author, the place of study, the year of publication, the objectives, the methodology employed, and the primary results and conclusions relevant to the central question of the scoping review. The organization of the articles was conducted in accordance with the methodological recommendations of Peters et al. [27].

The review identified three overarching themes pertaining to the support requirements of informal caregivers in PC, underscoring the imperative to avert caregiver burden and emphasizing the pivotal role of the FD in this context. The identified themes are as follows: firstly, factors that contribute to informal caregiver burden; secondly, strategies adopted by the FD to reduce informal caregiver burden; and thirdly, challenges and barriers that hinder the FD’s involvement in supporting these caregivers.

### 3.2. Factors That Contribute to the Informal Caregiver’s Burden

#### 3.2.1. Worsening of the Patient’s Symptoms

The exacerbation of psychological symptoms, including tension, anguish, and depression, in patients with PC has been associated with a significant increase in the burden on informal caregivers. Furthermore, as the disease progresses, the intensification of symptoms not only accentuates the patients’ state of suffering, but also amplifies the psychological impact on caregivers, who face the dual challenge of simultaneously managing the patients’ physical and emotional needs and the responsibility of providing ongoing care. The study by Krug et al. highlights that the emotional deterioration of patients, especially in the more advanced stages of the disease, is one of the main determinants of the emotional burden experienced by caregivers, compromising their well-being. Another study demonstrated that the emotional suffering of caregivers, intensified by anticipatory grief, has repercussions on multidimensional fatigue, both physical and emotional, exacerbating the burden during caregiving [28,29,30,31,32,33,34,35].

#### 3.2.2. Growing Functional Dependence of the Patient

As demonstrated by the data, the progression of the disease and the consequent functional dependence of patients in carrying out activities of daily living significantly increase the burden on caregivers. This growing reliance necessitates greater time and effort, both physically and psychologically, resulting in a substantial impact on the quality of life of informal caregivers [29].

#### 3.2.3. Challenging Behavior and Insecure Situations

The presence of challenging behaviors, such as agitation and mental confusion, as well as feelings of insecurity in the home environment, has been identified as a critical factor contributing to increased caregiver burden. These behaviors necessitate constant attention and frequently surpass the caregivers’ capacity to provide adequate care, prompting them to reevaluate the sustainability of home care. The aforementioned challenges have been shown to exacerbate the physical and emotional strain on caregivers, as evidenced by the study by de Jong et al. [29].

#### 3.2.4. Reconciling Care with Professional Activity and Other Responsibilities

Caregivers who are required to balance and manage caring for a sick family member with their professional and familial responsibilities face an even greater burden. The need to balance multiple tasks becomes exhausting as the disease progresses, especially for those who do not live with the patient. This exacerbates the emotional and physical pressure resulting from constant vigilance and insecurity about the patient’s condition [28].

#### 3.2.5. Impact on Social and Professional Life

The repercussions of caregiving have been demonstrated to exert a direct influence on the social interactions and professional life of caregivers, giving rise to social isolation and an increase in absenteeism from work. This impairment of the balance between personal and professional life contributes to an increase in the emotional, physical, and psychological overload of informal caregivers [28].

#### 3.2.6. Communication with Health Professionals

The absence of clarity and precision in interactions with health professionals is a significant contributing factor to the exacerbation of caregivers’ emotional overload. The frustration resulting from ineffective communication, coupled with a perceived lack of empathy on the part of professionals, intensifies the emotional distress experienced by caregivers. This, in turn, has a detrimental effect on caregivers’ ability to comprehend information regarding their patients’ clinical conditions, as well as their capacity to make informed decisions about the care they should provide [28].

#### 3.2.7. Socio-Economic Inequalities

Socio-economic inequalities have been shown to directly influence healthcare access, thereby contributing to the burden on caregivers. Families with greater financial resources have greater access to health services, including consultations with doctors working in the private sector and the possibility of hiring services from professional caregivers. They are also able to purchase essential devices and accessories for self-care, as well as food needed to comply with specific diets. Conversely, individuals with constrained economic resources encounter significant challenges in addressing fundamental care and treatment requirements, which can intensify their suffering. These inequalities further compromise access to healthcare, resulting in a more adverse disease process for patients in situations of financial vulnerability. In this regard, socio-economic status has been shown to amplify the impact of suffering, thereby contributing to disparities in care and well-being experienced by different groups. This underscores the need for a critical examination of equity policies in accessing healthcare [28].

### 3.3. FD Strategies to Support Informal Caregivers with an Emphasis on Relieving the Burden Experienced by Caregivers

#### 3.3.1. Holistic Care Model

A shared aspiration for a holistic care model was identified, encompassing emotional, social, and psychological support, in conjunction with medical treatment. This integrated approach is regarded as being fundamental to meeting the diverse needs throughout the disease process. Recognizing the needs of caregivers is crucial to optimizing the care provided to patients in the context of PC. It is therefore imperative that informal caregivers receive adequate support to prevent complications, including early detection of problems, monitoring of daily care tasks, and access to external support. The implementation of mental health programs, health education programs, and the research and provision of appropriate services are strategies that can significantly contribute to reducing the burden associated with caregiver home care [29,30,34].

It should be noted that the implementation of regular assessments of caregiver burden and the provision of early therapeutic interventions have been suggested as effective strategies for reducing the negative impact on caregivers [29].

#### 3.3.2. Relationships of Trust and Communication

The establishment of robust relationships and an appreciation for patients’ personal and familial dynamics were identified as paramount. The majority of caregivers articulated that FDs were regarded as approachable health professionals and astute listeners, thereby fostering conducive environments for open dialogue. Furthermore, FDs were found to demonstrate sensitivity to confidentiality issues, addressing caregivers’ concerns on an individual basis [34].

#### 3.3.3. Accessibility to Healthcare

According to the studies found, the majority of informal caregivers have a regular FD, with around 93% mentioning consultations in the last year, although most of these consultations were not related to their role as a caregiver. With regard to the accessibility of healthcare, the majority of patients and caregivers expressed satisfaction with the availability of FDs, especially in emergency situations. However, geographical distance can pose a challenge to face-to-face access for some caregivers. The importance of flexibility and the planning of medical schedules in ensuring continuity of care has been identified in the literature [32,34].

One of the studies highlights the potential of communication technologies to enhance interpersonal interaction and the flow of information during end-of-life care. Teleconsultations are therefore considered a promising tool for optimizing access to PC, promoting more frequent and effective communication between FDs, patients, and informal caregivers [31].

#### 3.3.4. Coordination and Communication Between Health Services

The data obtained underlines the significance of coordination and communication between primary and secondary healthcare providers. The objective of this coordination is to minimize discrepancies in information and to promote continuity of care. Furthermore, the empowerment of caregivers to make decisions is also promoted [29].

#### 3.3.5. Continuity of Care and Support After Death

Finally, the continuity of relationships between FDs and caregivers is imperative to ensure the provision of quality care, particularly in contexts of advanced illness. The provision of additional support to manage emotional pain and the grieving process post-mortem was identified as a key element to consider [29].

### 3.4. Challenges and Barriers Requiring FD Involvement

#### 3.4.1. Interprofessional Collaboration and Communication

The studies by Johnson et al. and Tan et al. emphasized the significance of interprofessional collaboration and effective communication between FDs, patients, and caregivers in order to ensure coordinated PC care. Despite this, primary care doctors expressed concerns about the quality of information sharing with secondary care, highlighting the need to stay informed about treatment and possible adverse effects. The absence of effective multidisciplinary communication can result in a fragmented approach, thereby impeding family practitioners’ capacity to make informed decisions and provide adequate support to patients and caregivers, which in turn can exacerbate their burden [33,34].

It is imperative to underscore that an open dialogue concerning prognosis and disease progression is indispensable for effective care planning [33].

#### 3.4.2. Knowledge About PCs

It is evident that both health professionals and society in general have insufficient knowledge about PC, which is generally only associated with terminally ill patients. Through analysis of focus groups, Tan et al. found agreement among patients, caregivers and clinicians that there was a need to educate the public so that everyone, inside and outside the health system, is aware that palliative care does not mean the end of life or imminent death. Education and training on the importance of this care at all stages of the disease can empower patients and their families more effectively, reducing the emotional burden [33].

#### 3.4.3. Domiciliary Care

In the article by Tan et al., it is stated that many patients reported difficulties in finding a FD willing to continue their care in the community as their palliative care needs increased. This reluctance was often due to discomfort or lack of expertise in managing palliative care at home, especially as patients become more housebound. This creates a gap in care, leaving patients without the support they need at a critical time in their lives. The efficacy of home care in facilitating enhanced access to the health system and optimized quality of care has been demonstrated by the implementation of a multidisciplinary, team-centered model. The successful implementation of this model is contingent upon the investment in home care teams with PC skills, thereby ensuring the provision of adequate support for patients and their families [33].

#### 3.4.4. Safety Within the Domestic Environment

Informal caregivers and health professionals emphasize the importance of structural factors. One of the perceived challenges affecting patient safety is access to and installation of suitable equipment. The FD plays a pivotal role in evaluating and coordinating protective measures, as well as in coordinating services and support within the community, when required [28].

## 4. Discussion

This review has highlighted several factors that contribute to the burden of informal caregivers in the context of PC. Research, including that of Krug et al., has demonstrated that the deterioration of patients’ clinical conditions, particularly the exacerbation of symptoms and the decline in emotional well-being, directly contributes to an increase in perceived caregiver burden. Patients’ emotional distress, exacerbated in the terminal phase, has been shown to increase the physical workload of caregivers, as well as having a negative impact on their mental health [35].

The caregiving process is further complicated by the occurrence of challenging behaviors and the increased dependence of patients, as demonstrated in the study by de Jong et al. In such cases, caregivers have reconsidered their ability to maintain care at home, especially when the burden exceeds their physical and emotional capacity. The combination of caregiving responsibilities with work and family demands increases the physical and emotional strain on caregivers. This situation is further exacerbated by a lack of adequate support and uncertainty about crisis management in the absence of the caregiver [29].

Another relevant factor to mention is the impact of anticipatory grief, highlighted in the study by Doubova et al., which contributes to the emotional suffering of caregivers as the illness of the family member being cared for progresses. In this sense, this circumstance, combined with physical fatigue and emotional exhaustion, intensifies the multidimensional burden that affects the personal, professional, and financial spheres of informal caregivers [28]. Research by Stenberg et al. and Kent et al. indicates that caregivers experience considerable impacts on their physical and mental health, including extreme fatigue, sleep disturbances, and anxiety [4,10]. The extant literature also emphasizes the relationship between the progression of the patient’s illness and the increasing burden on caregivers, as evidenced by the works of van Ryn et al. and Grunfeld. These findings serve to reinforce the results of the present review, which demonstrated that the worsening of patient symptoms and the increasing functional dependence of the patient are critical factors contributing to the burden experienced by caregivers [11,13].

It should be noted that ineffective communication with health professionals, especially the FD, exacerbates this process. In this sense, the lack of empathy and clear information about the clinical condition of the sick family member is an obstacle to the well-being of the patient-informal caregiver dyad. Therefore, this phenomenon highlights the need for a more patient- and caregiver-centered approach that integrates effective and empathic communication as essential components of clinical practice, with the aim of alleviating suffering and improving health outcomes [28].

Socio-economic inequalities also play a crucial role in the experience of this process of suffering and distress, as limited access to quality health services and essential care resources increases the burden on caregivers with fewer financial resources [28].

However, a distinctive aspect of this review is its analysis of the role of FDs in supporting informal caregivers, a topic that remains underexplored in the literature. Most existing studies focus on the burden of informal caregiving itself, without looking at the specific interventions provided by FDs. This review therefore helps to fill this gap by highlighting support strategies and challenges faced by FDs in supporting caregivers.

In accordance with this line of thought, it can be posited that this review underscores the necessity for a more equitable and integrated approach, encompassing not only the provision of support for the patient’s needs but also the adoption of strategies to alleviate the multidimensional burden on caregivers. The investment in emotional, financial and logistical support programs, as well as the implementation of appropriate support policies, is imperative for the enhancement of the quality of care [28].

With regard to the strategies adopted by the FD, the conclusions of the study by Krug et al. underscore the significance of their proactive action in the continuous assessment of patients’ needs and the level of burden faced by caregivers, with a view to enabling early intervention. In PC, the enablement of patients to receive care in the comfort of their own homes during the final stages of life is predicated on the recognition that concerns, needs and the burden of informal care must be accorded equal priority, given their inherent interconnectedness. It is imperative to acknowledge that the caregiver’s burden has the potential to adversely impact their own well-being and the quality of care imparted. The integration of PC with a focus on managing and relieving the patient’s physical and emotional symptoms has been identified as a strategy to alleviate the burden on caregivers [35].

The study by Roberts and Struckmeyer [36] highlights the importance of caregiver resilience strategies and respite programs as fundamental mechanisms to reduce caregiver burden. Our study confirms that the implementation of structured support can reduce the emotional burden of informal caregivers.

Furthermore, Aabom and Pfeiffer [37] discuss the reluctance of patients to seek support from FDs, highlighting the need for more active involvement of these professionals. This observation is consistent with the challenges identified in our review, particularly the lack of effective communication between caregivers and health professionals.

The study by Edwards et al. [38] complements this perspective by highlighting the importance of health literacy in the interaction between caregivers and health services. The inclusion of educational and informational support for caregivers could therefore mitigate some of the barriers identified.

Studies such as those by Doubova et al. and Aubin et al. suggest that psychosocial and educational support programs have a significant impact on reducing caregiver stress and exhaustion. The implementation of structured interventions, such as telephone support and teleconsultation, has also proven to be an effective alternative to ensure follow-up and support for informal caregivers, as shown by Ülgüt et al. [28,31,32].

In light of the aforementioned points, it can be concluded that the provision of adequate and prompt support is of paramount importance in enhancing the resilience of caregivers. This support ought to encompass interventions by FD, home care professionals, and the broader social network. Moreover, the organization of day care services and the scheduling of care responsibilities can significantly contribute to the capacity of informal caregivers to provide care. The findings of this study are corroborated by other research, which highlights the importance of additional support and structured forms of respite care for sustaining the resilience of family caregivers, thus ensuring continuity of care in the home environment [29,36].

In the de Jong et al. study, the resilience of informal caregivers played a crucial role, according to the testimonies of the caregivers themselves and the health professionals interviewed. While resilience was not always explicitly mentioned, it was frequently described in terms of “being able to cope” and “being able to keep up” with the demands of care. FDs emphasized the importance of assessing the resilience of the informal caregiver as a determining factor in supporting the decision to continue caring for the patient at home, thereby reinforcing the notion that the caregiver’s ability to cope with challenges is essential to ensure the viability of care at home [29].

The review revealed that insufficient inter-professional collaboration and inadequate information sharing between primary and secondary healthcare represent significant barriers to the effective involvement of FDs in end-of-life care. The included studies demonstrated that the lack of regular information about patients’ clinical status and ongoing treatments prevents FDs from making informed decisions, which limits the support they can offer to patients and their caregivers. This communication gap has been shown to contribute to a fragmented approach to care, which in turn places additional burdens on caregivers [33,34].

Following on from this, this review emphasizes the importance of transparent and honest communication by FDs. Objective and accessible explanations of medical concepts and terminology are valued, with an emphasis on the need for simple and comprehensible language. Patients have highlighted the importance of having an intermediary to help them interpret medical terminology within the specific context of their condition. Patients and caregivers underscored the significance of being listened to during consultations with FDs, a fundamental component of the doctor-patient-caregiver triad. From a physician’s perspective, the ability to communicate effectively, the process of simplifying medical information, and aiding in decision-making are all considered to be integral components of their role in end-of-life care. This process entails the clarification of complex medical terminology, thereby facilitating a comprehensive understanding of the disease, its progression, and the proposed treatment by patients and caregivers [34].

The specialty of FM is predicated on effective communication, which is an essential component of end-of-life care. Therefore, the communicative competence of FDs in this context goes beyond the mere transmission of clinical information, as it implies the ability to communicate clearly, honestly and empathetically, adapting to the emotional and cognitive needs of patients and informal caregivers, who are facing a time of great vulnerability. It is therefore vital that they are able to explore and explain complex and challenging concepts in an accessible and understandable manner, especially given that patients and caregivers often have difficulty understanding medical terminology and the workings of the health system and care plan during periods of high stress and exhaustion. In this guideline, clear communication facilitates effective understanding, promotes trust, reduces uncertainty and contributes to informed, free and enlightened decision-making, which are indispensable conditions for quality PC [37,38].

The importance of the FD’s ongoing involvement should be noted, especially in rural areas, where proximity and knowledge of the needs and reality of patients and their caregivers is valued. Clear and accessible communication, coupled with time dedicated to consultations, has been identified as essential for fostering trust and facilitating the process of informed decision-making [34].

It is therefore imperative that informal caregivers receive support to prevent complications, including the early detection of problems, daily prevention, monitoring of daily care tasks, and access to external support. These measures are pivotal in reducing work overload. Furthermore, it is essential that caregivers develop support strategies aimed at reducing tension and emotional burden. The implementation of programs aimed at mental health, continuing education, research and the provision of appropriate services are all measures that can significantly contribute to reducing the burden associated with home care on the part of the caregiver. A comprehensive and integrated approach is therefore fundamental to addressing the needs of caregivers and enhancing the quality of care provided [30].

The study by Tan et al. revealed that both the medical community and society in general still have the perception that PC is restricted to end-of-life care, rather than a continuous approach to improving quality of life throughout the illness. The training and education of FDs in the area of PC can reduce this barrier and improve the preparation of patients and caregivers, allowing for more effective advance planning [33].

The study also underscores the potential of teleconsultations as a functional instrument to enhance access to primary care and to facilitate regular interaction between FDs, patients and informal caregivers. However, it is important to note that these consultations may be subject to inherent limitations and challenges. A key challenge pertains to the necessity of conducting a comprehensive clinical evaluation remotely, as the absence of physical interaction impedes the FD’s capacity to discern significant signs and symptoms. Furthermore, the utilization of teleconsultations necessitates that both patients and caregivers possess the necessary technological aptitude and familiarity with its application, a factor that can impose limitations, particularly among older demographics, economically disadvantaged populations, or those residing in areas with constrained technological access. Conversely, teleconsultations have the capacity to enhance home care by facilitating regular monitoring and offering a form of rapid intervention in emergent situations, thereby complementing, rather than replacing, the importance of face-to-face consultations. In this regard, they can be regarded as a potentially valuable addition to the existing healthcare framework, rather than a substitute, for the essential personalized and comprehensive care that is provided through face-to-face interactions [31].

One of the studies has some limitations that should be considered. Firstly, the methodological approach adopted, characterized as a single interpretative structural modelling model, which may restrict the breadth and depth of the results obtained. Secondly, the small sample size, which limits the ability to accurately represent all groups of informal caregivers, which may compromise the generalizability of the conclusions. In addition, the in-depth interviews were carried out using a fixed format questionnaire, which may have introduced bias into the data collection. Thirdly, the results are based on the perceptions of the participants, which means that they cannot be reproduced in other contexts or populations. Therefore, it is recommended that future research considers using mixed methods and considers exploring different groups of informal caregivers, increasing the sample size [30].

A thorough analysis of the included studies has revealed significant gaps in the extant research. A preponderance of the studies is of a qualitative nature. This imbalance may be attributed to the suitability of qualitative methods for capturing the subjective perspectives of informal caregivers and FDs. It is also important to note that many of the qualitative studies had small samples, which limits the extent to which the results can be generalized. This underscores the necessity for a greater volume of quantitative and mixed-methodology studies, which are designed to provide more robust and detailed data, thereby facilitating a more profound comprehension of the subject under investigation.

We can infer that the literature includes few studies that link the three themes addressed: the role of the FD, PC, and the burden of the informal caregiver. Although there is a vast body of literature highlighting the importance of the FD in PC, the studies tend to focus on the accessibility, continuity, and quality of care provided to patients, neglecting to systematically explore the relationship between the actions of these professionals and the prevention of informal caregiver overload. Most studies identify the importance of the FD in coordinating care, providing emotional support, and communicating effectively with patients, caregivers, and health professionals. However, the correlation between these interventions and the reduction of caregiver burden is mentioned tangentially, without being the subject of central research or rigorous quantification.

This gap in the literature highlights the necessity for further research investigating the extent to which FDs’ practices and interventions have an impact on preventing informal caregiver burden in the context of PC.

## 5. Conclusions

The family doctor plays an essential role in the provision of palliative care and support to informal caregivers, thereby helping to reduce their emotional, physical and financial burden. Continuity of care, building relationships of trust and coordinating health resources are foundational aspects for the optimization of the quality of life of patients and their caregivers. However, significant challenges remain that require the implementation of effective strategies and a sustained commitment from the organizations responsible for promoting adequate support. Future research should focus on improving support policies for informal caregivers and the ongoing training of health professionals, ensuring a more sustainable and humanized system.

## Figures and Tables

**Table 1:** Articles included in the scoping review.

Title|Authors|Year|Country	Objectives	Methodology	Discussion/Conclusions
Dimensions of suffering and the need for palliative care: experiences and expectations of patients living with cancer and diabetes and their caregivers in Mexico—a qualitative study [28]. Doubova et al. 2023—Mexico	Explore the meaning and experiences of suffering, as well as the forms of relief, in informal caregivers and patients diagnosed with cancer or diabetes.	Qualitative study Patients (group 1) and informal caregivers (group 2) (*n* = 68) Group 1 = 33 Group 2 = 35	The multifaceted nature of severe health-related suffering poses a considerable challenge to health systems, obliging them to provide holistic, high-quality PC. Policies aimed at expanding access to PC should consider integrating it into primary care, ensuring adequate training for professionals and safe access to healthcare, as well as redesigning services to meet the needs of the patient and caregiver.
What Facilitates or Hampers Living at Home With Advanced Dementia Until the End of Life? A Qualitative Study Using Retrospective Interviews Among Family Caregivers, General Practitioners, and Case Managers [29]. de Jong et al. 2023—Netherlands	Understand the factors that make it easier or more difficult for people with advanced dementia to stay at home until the end of their lives.	Qualitative study Informal caregivers (group 1), family doctors (group 2), and/or case managers (group 3) (*n* = 12) Group 1 = 11 Group 2 = 2 Group 3 = 9 A series of interviews were conducted with the bereaved informal caregiver, the family doctor, and the case manager who was most involved in caring for the person with dementia.	Patients diagnosed with dementia who remained at home until the conclusion of their lives frequently exhibited caregivers who received sufficient support from healthcare professionals and their social network. The integration of case management with the continuous process of advance planning for personalized care has been demonstrated to enhance the probability of the patient’s environment aligning with their values and needs. Patients diagnosed with dementia who opted to remain in their homes until the conclusion of their lives frequently exhibited a higher degree of support from professional caregivers and their social networks. The integration of case management with the ongoing process of personalized advance care planning has been demonstrated to enhance the congruence between the home environment at the end of life and the values and needs of both the patient and family caregivers.
Listening to Caregivers’ Voices: The Informal Family Caregiver Burden of Caring for Chronically Ill Bedridden Elderly Patients [30]. Mamom J, Daovisan H. 2022—Thailand	Investigate the burden of informal caregivers in the process of caring for bedridden elderly people with chronic illnesses.	Qualitative study Informal caregivers (*n* = 30) Interviews based on the total interpretative structural model with caregivers of bedridden elderly people with chronic illnesses.	The burden of informal caregivers in PC has been associated with various responsibilities, such as daily workload, follow-up care, daily tasks, stress inherent in care, strategies adopted, monitoring, financial impact, and patient support. Interventions focused on redistributing responsibilities, external monitoring, adequate support and planning daily tasks can help reduce this burden.
Experiences and support needs of informal long-distance caregivers at the end of life: a scoping review [31]. Ülgüt et al. 2021—Germany	Carry out a literature review on the experiences and needs of informal caregivers at a distance.	Scoping review *n* = 21 articles.	Five themes were identified: (1) geographical distance as a barrier to caregiving; (2) communication difficulties and the role of video and telephone calls; (3) the burdens and benefits associated with long-distance caregiving; (4) interaction and conflicts with local caregivers; (5) support needs and expectations for long-distance caregivers.
A randomized clinical trial assessing a pragmatic intervention to improve supportive care for family caregivers of patients with lung cancer [32]. Aubin et al. 2021—Canada	Evaluate the feasibility and preliminary effects of an intervention aimed at improving supportive care for family caregivers.	Randomized clinical trial Informal caregivers (*n* = 109) Group 1 = 54 (intervention) Group 2 = 55 (regular care) Systematic screening of caregivers’ distress and assessment of their problems in the first few months after the family member’s cancer diagnosis, with follow-up every two months, using the Hospital Anxiety and Depression Scale and the Edmonton Symptom Assessment Scale. Liaison with the family doctor of caregivers who reported a high level of distress, with the aim of involving them in the provision of supportive care.	FDs were invited to contribute to caregiver support, as emotional support is recognized as an essential part of their role in cancer care. Their long-standing relationship with patients and familiarity with their social context put them in a strategic position to provide this support, and it was assumed that their actions could favorably influence the emotional stress faced by caregivers. Although the intervention was not considered fully effective, some of its aspects were positively perceived by informal caregivers. Given that many of these caregivers face high levels of distress, it becomes crucial to develop an improved intervention that more effectively addresses their specific needs.
Family physicians supporting patients with palliative care needs within the patient medical home in the community: an appreciative inquiry study [33]. Tan et al. 2021—Canada	To identify the essential components to more effectively support patients and their families with PC needs, with the aim of guiding changes in the system and empowering FDs in the provision of this care.	Qualitative study Family doctors (group 1), informal caregivers (group 2), patients (group 3) and home care team (group 4) (*n* = 12) Group 1 = 18 Group 2 = 8 Group 3 = 1 Group 4 = 26 FDs took part in semi-structured interviews, based on a script designed to assess and explore the current landscape of FM and the provision of PC. In addition, separate focus groups were used to discuss the perspectives and experiences of patients, bereaved informal caregivers and home-based PC teams.	The central concept was the need to improve communication and promote a collaborative relationship between all care providers, with a focus on both the patient and informal caregivers. The relationship between the FD and the patient must be preserved and encouraged by all those involved in the care process, while the healthcare system needs greater flexibility to respond more effectively to patients’ needs. These principles should be applied in a context where both patients and caregivers need more information about the benefits of PC, alongside an increase in public dialog on the subject of death. Key areas have been identified to optimize the collaboration of the multidisciplinary team in PC, with the aim of improving the care pathway for the patient and the caregiver. Strengthening the relationship of trust between the FD and the patient is fundamental for effective care and the satisfaction of those involved.
End-of-life care in rural and regional Australia: Patients’, carers’ and general practitioners’ expectations of the role of general practice, and the degree to which they were met [34]. Johnson et al. 2020—Australia	To investigate the characteristics of FM specialty practice in rural settings that exemplify ideal PC, according to the perspective of people diagnosed with cancer, informal caregivers, and FDs. To assess the extent to which patients and caregivers felt that the care they received corresponded to these ideal characteristics.	Qualitative study Family doctors (group 1), informal caregivers (group 2), patients (group 3) (*n* = 13) Group 1 = 4 Group 2 = 3 Group 3 = 6	Seven key characteristics were identified for optimizing end-of-life care: (1) commitment and accessibility; (2) development of therapeutic relationships; (3) efficient communication; (4) psychosocial support; (5) competent symptom management; (6) integrated care coordination; (7) recognition of caregivers’ needs. The majority of general practitioners addressed these dimensions consistently and in line with best practice.
Correlation between patient quality of life in palliative care and burden of their family caregivers: a prospective observational cohort study [35]. Krug et al. 2016—Germany	To identify the correlation between the quality of life of patients undergoing CP and the burden experienced by their family caregivers.	Prospective cohort study Family doctors (group 1) and informal caregivers (group 2) (*n* = 147) Group 1 = 47 Group 2 = 100 The quality of life of cancer patients undergoing PC, in the context of primary health care, was assessed using the Quality of Life Questionnaire Core 15 Palliative Care. Informal caregivers reported the burden associated with providing support to the sick family member, assessed using the short version of the Burden Scale for Family Caregivers.	The patients’ dyspnea, depression, and anxiety symptoms had a significant impact on the caregivers’ perception of burden, despite the fact that these symptoms are manageable with appropriate interventions. These results corroborate the importance of regular and systematic monitoring of patients’ needs, including assessment of the impact these symptoms have on caregivers. Through a proactive and continuous approach, FM teams can anticipate and reduce the risk of caregiver burden, allowing for timely and more effective interventions in end-of-life care management.

## Data Availability

The original contributions presented in this study are included in the article. Further inquiries can be directed to the corresponding author.

## Abbreviations

The following abbreviations are used in this manuscript:

BMJ	British Medical Journal
FD	Family doctor
FM	Family medicine
MeSH	Medical Subject Headings
NICE	National Institute for Health and Care Excellence
PC	Palliative care
PRISMA-ScR	Preferred Reporting Items for Systematic Reviews and Meta-Analyses extension for Scoping Reviews
